# The brain–bone axis: unraveling the complex interplay between the central nervous system and skeletal metabolism

**DOI:** 10.1186/s40001-024-01918-0

**Published:** 2024-06-08

**Authors:** Haojun Shi, Min Chen

**Affiliations:** Faculty of Chinese Medicine and State Key Laboratory of Quality Research in Chinese Medicines, Macau University of Science and Technology, Macau, Macau SAR China

**Keywords:** Brain–bone axis, Neuropeptides, Neurotransmitters, Bone-derived factors

## Abstract

The brain–bone axis has emerged as a captivating field of research, unveiling the intricate bidirectional communication between the central nervous system (CNS) and skeletal metabolism. This comprehensive review delves into the current state of knowledge surrounding the brain–bone axis, exploring the complex mechanisms, key players, and potential clinical implications of this fascinating area of study. The review discusses the neural regulation of bone metabolism, highlighting the roles of the sympathetic nervous system, hypothalamic neuropeptides, and neurotransmitters in modulating bone remodeling. In addition, it examines the influence of bone-derived factors, such as osteocalcin and fibroblast growth factor 23, on brain function and behavior. The therapeutic potential of targeting the brain–bone axis in the context of skeletal and neurological disorders is also explored. By unraveling the complex interplay between the CNS and skeletal metabolism, this review aims to provide a comprehensive resource for researchers, clinicians, and students interested in the brain–bone axis and its implications for human health and disease.

## Introduction

The traditional perspective of the skeletal system and the central nervous system (CNS) as distinct entities with separate functions within the human body has been challenged by recent advancements in research, leading to the recognition of a new field of study known as the brain–bone axis [1, 2]. This emerging area of investigation reveals a bidirectional communication network between the brain and bone, thereby questioning the conventional understanding of the skeleton solely as a structural support system and calcium reservoir [3, 4].

This comprehensive review aims to provide a detailed exploration of the current state of knowledge surrounding the brain–bone axis. The manuscript is structured to first discuss the neural regulation of bone metabolism, focusing on the roles of the sympathetic nervous system, hypothalamic neuropeptides, and neurotransmitters in modulating bone remodeling. Next, it examines the influence of bone-derived factors, such as osteocalcin and fibroblast growth factor 23, on brain function and behavior. The review then explores the therapeutic implications and potential clinical applications of targeting the brain–bone axis in the context of skeletal and neurological disorders. Finally, it identifies current gaps in understanding and proposes future directions for research in this rapidly evolving field.

The brain–bone axis encompasses a multitude of signaling pathways, neuroendocrine factors, and molecular mediators that orchestrate the crosstalk between the CNS and skeletal metabolism. The CNS exerts significant control over bone remodeling through various neural pathways, including the sympathetic nervous system [5, 6], hypothalamic neuropeptides [7, 8], and neurotransmitters [9]. Conversely, bone-derived factors, such as osteocalcin [10, 11] and fibroblast growth factor 23 (FGF23) [12, 13], have been shown to influence brain function and behavior, crossing the blood–brain barrier and modulating cognitive processes, memory, and mood.

The clinical implications of the brain–bone axis are far-reaching, spanning a wide range of skeletal and neurological disorders. Dysregulation of the signaling pathways involved in this complex system may contribute to the pathogenesis of conditions such as osteoporosis, fractures [14, 15], Alzheimer’s disease (AD) [16, 17], and depression [18, 19]. Unraveling the intricacies of the brain–bone axis holds promise for the development of novel diagnostic tools, therapeutic interventions, and preventive strategies that target both skeletal and neurological health.

This review offers a comprehensive analysis of the intricate relationship between the central nervous system (CNS) and skeletal metabolism, serving as a valuable reference for individuals involved in research, clinical practice, and academia within this field of study. Delving into the concept of the brain–bone axis not only enhances understanding of the complex interconnections between seemingly unrelated physiological systems in the human body but also presents novel opportunities for addressing various skeletal and neurological disorders through advancements in prevention, diagnosis, and treatment strategies.

## Neural regulation of bone metabolism

Neural regulation of bone metabolism involves complex interactions between various components of the nervous system and bone tissue. This section explores the roles of the sympathetic nervous system, parasympathetic nervous system, hypothalamic neuropeptides, and neurotransmitters in modulating bone remodeling.

### Sympathetic nervous system

**Fig. 1:**
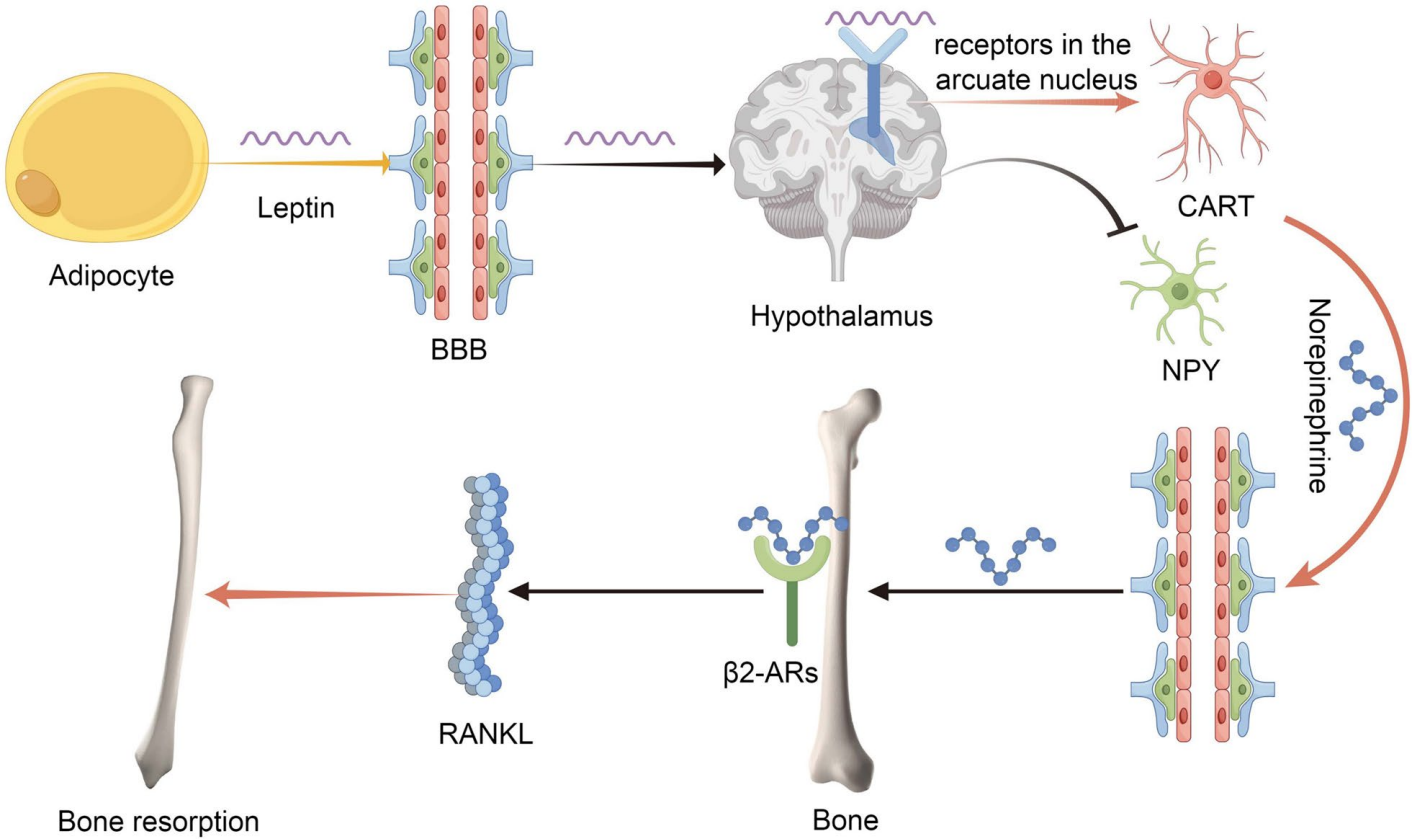
The leptin–hypothalamus–SNS–bone axis

The sympathetic nervous system (SNS) plays a pivotal role in the regulation of bone metabolism, serving as a key mediator of the central control of bone remodeling (Fig. 1). The SNS innervates bone tissue, with sympathetic nerve fibers directly contacting osteoblasts and osteoclasts, the cells responsible for bone formation and resorption, respectively.

Norepinephrine, the primary neurotransmitter of the SNS, binds to β2-adrenergic receptors (β2-ARs) expressed on osteoblasts, leading to a cascade of signaling events that ultimately suppress bone formation and favor bone resorption [5]. Activation of β2-ARs on osteoblasts results in the down-regulation of osteoblast proliferation and differentiation, while simultaneously up-regulating the expression of receptor activator of nuclear factor-κB ligand (RANKL) [20]. RANKL, in turn, stimulates osteoclast differentiation and activity, thereby promoting bone resorption [21].

The central regulation of bone mass through the SNS is exemplified by the leptin–hypothalamus–SNS–bone axis [6]. Leptin, an adipocyte-derived hormone, crosses the blood–brain barrier (BBB) and binds to its receptors in the arcuate nucleus of the hypothalamus, leading to the activation of cocaine- and amphetamine-regulated transcript (CART) neurons and the suppression of neuropeptide Y (NPY) neurons [22]. This hypothalamic response results in increased sympathetic outflow to the skeleton, promoting bone loss through the activation of β2-ARs on osteoblasts [23].

The role of the sympathetic nervous system (SNS) in bone metabolism is supported by clinical evidence, as evidenced by studies linking conditions marked by heightened sympathetic activity, such as reflex sympathetic dystrophy and postmenopausal osteoporosis, with decreased bone mineral density [24, 25]. In addition, pharmacological interventions that target the SNS, such as β-blockers, have been demonstrated to lower fracture risk in epidemiological studies, underscoring the potential therapeutic benefits of manipulating sympathetic signaling in the realm of skeletal disorders [26, 27].

### Parasympathetic nervous system

While the role of the SNS in bone metabolism has been well established, the involvement of the parasympathetic nervous system (PNS) in skeletal homeostasis has received less attention. However, emerging evidence suggests that the PNS also plays a role in the regulation of bone remodeling, counterbalancing the catabolic effects of the SNS [28].

The PNS innervates bone tissue, with cholinergic nerve fibers expressing the neurotransmitter acetylcholine (ACh) in close proximity to bone cells [29]. ACh has been shown to exert anabolic effects on the skeleton by binding to nicotinic acetylcholine receptors (nAChRs) expressed on osteoblasts, promoting their proliferation and differentiation [30].

In vivo studies have demonstrated that vagal nerve stimulation (VNS), which activates the PNS, leads to increased bone mass in mice [31]. This effect was mediated by the binding of ACh to α7 nAChRs on osteoblasts, resulting in enhanced bone formation. Conversely, pharmacological or surgical disruption of vagal signaling resulted in decreased bone mass and increased bone resorption [32].

The role of the PNS in bone metabolism is further supported by clinical observations of increased fracture risk in patients with conditions characterized by parasympathetic dysfunction, such as diabetes mellitus and inflammatory bowel disease [33, 34]. These findings suggest that an imbalance between the SNS and PNS may contribute to skeletal pathology, and highlight the potential therapeutic value of targeting the PNS in the management of bone disorders [35].

### Hypothalamic neuropeptides

The hypothalamus, a key regulator of homeostatic functions within the CNS, has emerged as a critical player in the central control of bone metabolism. Several hypothalamic neuropeptides have been implicated in the regulation of bone remodeling, acting through both direct and indirect mechanisms.

#### Leptin

Leptin, an adipocyte-derived hormone, plays a central role in energy homeostasis and has been shown to regulate bone mass through hypothalamic pathways [23]. Leptin crosses the blood–brain barrier and binds to its receptors in the arcuate nucleus of the hypothalamus, leading to the activation of anorexigenic neurons expressing pro-opiomelanocortin (POMC) and CART, while suppressing orexigenic neurons expressing NPY and agouti-related peptide (AgRP) [22].

The central effects of leptin on bone mass are primarily mediated through the SNS, with increased leptin signaling in the hypothalamus resulting in enhanced sympathetic outflow to the skeleton and subsequent activation of β2-ARs on osteoblasts, leading to decreased bone formation and increased bone resorption [6]. This leptin–hypothalamus–SNS–bone axis represents a critical pathway through which the CNS regulates bone homeostasis in response to changes in energy balance.

Interestingly, the peripheral effects of leptin on bone appear to be opposite to its central effects, with direct stimulation of osteoblast differentiation and bone formation, and inhibition of osteoclastogenesis [36]. These findings suggest that leptin regulates bone metabolism through a complex interplay of central and peripheral mechanisms, highlighting the need for further research to elucidate the specific pathways involved.

#### NPY

NPY is an orexigenic neuropeptide expressed in the hypothalamus and peripheral nervous system, and has been implicated in the regulation of bone metabolism through both central and peripheral mechanisms [37].

Central NPY signaling appears to have a catabolic effect on bone, with increased NPY expression in the hypothalamus associated with decreased bone mass and increased osteoclast activity [5]. These effects are thought to be mediated, at least in part, by NPY-induced suppression of osteoblast differentiation and increased RANKL expression, leading to enhanced osteoclastogenesis.

In contrast, peripheral NPY signaling has been shown to have an anabolic effect on bone, with NPY receptors (particularly Y1 and Y2 receptors) expressed on osteoblasts and osteoclasts [38]. In vitro studies have demonstrated that NPY directly stimulates osteoblast proliferation and differentiation while inhibiting osteoclastogenesis. Furthermore, mice lacking Y1 or Y2 receptors exhibit a low bone mass phenotype, highlighting the importance of peripheral NPY signaling in maintaining skeletal homeostasis [39].

#### CART

CART is an anorexigenic neuropeptide expressed in the hypothalamus and peripheral tissues, and has been implicated in the regulation of bone metabolism through both central and peripheral mechanisms [5].

Central CART signaling appears to have a catabolic effect on bone, with increased CART expression in the hypothalamus associated with decreased bone mass and increased osteoclast activity. These effects are thought to be mediated, at least in part, by CART-induced activation of the SNS, leading to increased β2-AR signaling in osteoblasts and subsequent suppression of bone formation and enhanced bone resorption [40].

Peripheral CART signaling, on the other hand, has been shown to have an anabolic effect on bone, with CART receptors expressed on osteoblasts and osteoclasts. In vitro studies have demonstrated that CART directly stimulates osteoblast differentiation and bone formation while inhibiting osteoclastogenesis. Moreover, mice lacking CART exhibit a low bone mass phenotype, highlighting the importance of peripheral CART signaling in maintaining skeletal homeostasis [5].

#### Neuromedin U

Neuromedin U (NMU) is a hypothalamic neuropeptide involved in the regulation of energy homeostasis and has recently been implicated in the control of bone metabolism [13]. NMU receptors, particularly NMUR1, are expressed in the hypothalamus and on osteoblasts, suggesting both central and peripheral roles in skeletal homeostasis.

Central NMU signaling appears to have a catabolic effect on bone, with increased NMU expression in the hypothalamus associated with decreased bone mass and increased osteoclast activity. These effects are thought to be mediated, at least in part, by NMU-induced activation of the SNS, leading to increased β2-AR signaling in osteoblasts and subsequent suppression of bone formation and enhanced bone resorption [41].

Peripheral NMU signaling, on the other hand, has been shown to have an anabolic effect on bone, with NMU directly stimulating osteoblast proliferation and differentiation through the activation of NMUR1 receptors [13]. In vitro studies have demonstrated that NMU promotes osteoblast survival and matrix mineralization while inhibiting osteoclastogenesis. Moreover, mice lacking NMUR1 exhibit a low bone mass phenotype, highlighting the importance of peripheral NMU signaling in maintaining skeletal homeostasis.

The results indicate that Hypothalamic Neuropeptides have a multifaceted impact on bone metabolism, with divergent effects on bone remodeling stemming from both central and peripheral signaling pathways. Additional investigation is required to elucidate the precise mechanisms driving these disparate outcomes and to investigate the therapeutic possibilities of modulating hypothalamic neuropeptides signaling in the realm of skeletal disorders.

#### Growth hormone releasing peptide (GHRP)

Growth hormone releasing peptide (GHRP) is a class of peptides with growth hormone (GH) releasing activity, mainly including Ghrelin, GHRP-2, and GHRP-6. GHRP is mainly synthesized and secreted by neuroendocrine cells, such as the arcuate nucleus of the hypothalamus [42]. GHRP is mainly synthesized and secreted by neuroendocrine cells such as the arcuate hypothalamic nucleus, and binds to the GH secretion receptor (GHS-R) to stimulate the pulsatile release of GH, thereby regulating bone metabolism and bone remodeling [43]. There is increasing evidence that GHRP and its signaling pathway are closely related to osteoporosis, osteoarthritis and other bone diseases [44]. In osteoporosis, GHRP can reduce bone loss by promoting the proliferation and differentiation of osteoblasts, inhibiting osteoclast activity, and improving bone marrow fat infiltration, etc. [45]. In osteoarthritis, GHRP can slow down the progression of the disease by protecting the chondrocytes, inhibiting the synovial inflammation, and improving the alteration of the subchondral bone, etc. [46]. The analogs of GHRP have shown good bone protection effects in animal models, and are expected to become innovative drugs for bone-derived diseases, and are expected to be the first drugs for bone diseases, and will be the most important drugs for bone diseases. GHRP analogs have shown good bone-protective effects in animal models and are expected to be innovative drugs for osteogenic diseases, but their long-term efficacy and safety need to be clinically proven. As an important regulator of the bone–brain axis, in-depth study of the regulation of bone metabolism by GHRP can help to understand the pathogenesis of osteogenic diseases from the perspective of neuroendocrine network and provide new ideas for the prevention and treatment of bone diseases [47].

#### Others

Corticotropin-releasing factor (CRF), thyrotropin-releasing hormone (TRH) and angiotensin II (AngII) are important components of the hypothalamic neuroendocrine system, and play key roles in bone metabolism homeostasis and the development of bone-originated diseases through the regulation of downstream hormone secretion and autonomic activity [48].

CRF is secreted by the paraventricular nucleus of the hypothalamus and is a core initiator of the stress response. Chronic stress can continuously activate CRF and cause osteoporosis through the following mechanisms: (1) CRF overexpression promotes hyperactivity of the hypothalamic–pituitary–adrenal axis, increases glucocorticoid secretion, inhibits osteoclasts, and accelerates bone resorption; (2) CRF stimulates the secretion of catecholamines through the sympathetic–adrenal axis, inhibiting bone formation and accelerating bone loss; (3) CRF inhibits gonadotrophin secretion through the secretion of pituitary ACTH, and accelerates bone loss; (4) CRF inhibits gonadotrophin secretion through the secretion of pituitary ACTH, and inhibits bone formation and bone loss. (iii) CRF inhibits gonadotropin secretion through pituitary ACTH secretion, lowering the level of sex hormones and aggravating bone loss [49]. Antagonizing CRF activity is expected to reduce the secondary osteoporosis induced by stress and depression.

TRH is secreted by the paraventricular nucleus of the hypothalamus and indirectly regulates bone metabolism by stimulating TSH secretion from the pituitary gland and promoting the synthesis and release of thyroid hormones [50]. Hyperfunction of the TRH/TSH axis can lead to hyperthyroidism, accelerated bone turnover, mainly bone resorption, and secondary osteoporosis, while hypoplasia of the TRH/TSH axis can lead to hypothyroidism, slower bone metabolism, and decreased bone mineral density. In recent years, it has been found that TRH can stimulate the activity of downstream Kiss1 neurons through the hypothalamic TRH receptor (TRHR), promote the function of gonadotropic axis and increase the level of sex hormones, thus directly affecting the bone metabolism [51]. TRH and its analogs can stimulate the differentiation of bone marrow mesenchymal stem cells to osteoblasts, promote bone formation, and alleviate the bone loss of de-virginized rats, which suggests that the signal of TRH may be a potential therapeutic target for osteoporosis. The signaling of TRH may be a potential therapeutic target for osteoporosis.

In addition to classical blood pressure and fluid regulation, AngII can also affect bone metabolism through the central nervous system. The paraventricular nucleus and arcuate nucleus of hypothalamus have AngII receptors, and AngII can stimulate the expression of CRF in hypothalamus, activate the HPA axis, and increase the level of glucocorticoid hormone through the AT1 receptor; on the other hand, it can activate the sympathetic nervous system and promote the release of catecholamines such as norepinephrine, and finally cause osteogenesis inhibition and bone resorption hyperactivity [52]. AngII antagonists can attenuate ovariectomy-induced osteoporosis by antagonizing central RAS activity. In addition, AngII may affect bone metabolism and fracture healing by regulating vascular tone and intraosseous perfusion. The over-activation of central RAS may be involved in the development of osteoporosis and other bone diseases through various pathways.

In conclusion, hypothalamic neuropeptides such as CRF, TRH and AngII are involved in the development of osteoporosis and other bone diseases by influencing the activities of downstream neuroendocrine axis and autonomic nervous system to regulate the balance of bone metabolism both systemically and locally, and the over-activation of CRF, the dysfunction of the TRH/TSH axis and the hyper-activation of the central RAS could be the endocrine mechanism of the development of osteoporosis, and the regulation of the relevant signaling pathways is expected to be a new strategy for the prevention and treatment of bone diseases [53]. The strategy of regulating the related signaling pathways is expected to bring new breakthroughs in the prevention and treatment of bone diseases. Meanwhile, there is a wide range of bidirectional regulation between bone metabolism and neuroendocrinology. In the future, it is necessary to deeply elucidate the role of the bone–brain axis and its abnormality in the development of bone diseases from a holistic and systemic point of view, and then formulate individualized diagnosis and treatment accordingly, so as to realize the precise prevention and treatment of osteogenic diseases.

### Neurotransmitters

In addition to neuropeptides, several neurotransmitters have been implicated in the regulation of bone metabolism, acting through both central and peripheral mechanisms. These neurotransmitters include serotonin, dopamine, glutamate, and γ-aminobutyric acid (GABA).

#### Serotonin

Serotonin (5-hydroxytryptamine, 5-HT) is a monoamine neurotransmitter involved in the regulation of mood, appetite, and sleep, and has been shown to play a complex role in the regulation of bone metabolism, with distinct central and peripheral effects [54].

Central serotonin signaling has been shown to have a catabolic impact on bone, as elevated serotonin levels in the brain are linked to reduced bone mass and increased osteoclast activity. It is believed that these effects are facilitated by serotonin-induced activation of the sympathetic nervous system (SNS), resulting in heightened β2-adrenergic receptor (β2-AR) signaling in osteoblasts, which subsequently inhibits bone formation and promotes bone resorption. Mice with a conditional deletion of tryptophan hydroxylase 2 (Tph2), the key enzyme in central serotonin synthesis, display a phenotype characterized by increased bone mass, further corroborating the detrimental role of serotonin in bone metabolism [55].

On the contrary, peripheral serotonin has been demonstrated to possess an anabolic influence on bone, as evidenced by the direct promotion of osteoblast proliferation and differentiation through the activation of 5-HT1B and 5-HT2B receptors by gut-derived serotonin. Mice lacking tryptophan hydroxylase 1 (Tph1), the key enzyme in peripheral serotonin production, display a reduced bone mass phenotype, underscoring the significance of peripheral serotonin signaling in the regulation of skeletal equilibrium [56].

#### Dopamine

Dopamine is a catecholamine neurotransmitter involved in the regulation of movement, reward, and motivation, and has been implicated in the regulation of bone metabolism through both central and peripheral mechanisms [57].

Central dopamine signaling appears to have a catabolic effect on bone, with increased dopamine levels in the brain associated with decreased bone mass and increased osteoclast activity. These effects are thought to be mediated by dopamine-induced activation of the SNS, leading to increased β2-AR signaling in osteoblasts and subsequent suppression of bone formation and enhanced bone resorption. Mice lacking dopamine transporter (DAT), which results in increased dopamine levels in the brain, exhibit a low bone mass phenotype, further supporting the catabolic role of central dopamine signaling in bone metabolism [58].

Peripheral dopamine signaling, on the other hand, has been shown to have an anabolic effect on bone, with dopamine receptors (particularly D2 receptors) expressed on osteoblasts and osteoclasts. In vitro studies have demonstrated that dopamine directly stimulates osteoblast proliferation and differentiation while inhibiting osteoclastogenesis through the activation of D2 receptors. Moreover, mice lacking D2 receptors exhibit a low bone mass phenotype, highlighting the importance of peripheral dopamine signaling in maintaining skeletal homeostasis [59].

#### Glutamate

Glutamate is the primary excitatory neurotransmitter in the CNS and has been implicated in the regulation of bone metabolism through both central and peripheral mechanisms [60].

Central glutamate signaling appears to have a catabolic effect on bone, with increased glutamate levels in the brain associated with decreased bone mass and increased osteoclast activity. These effects are thought to be mediated, at least in part, by glutamate-induced activation of the SNS, leading to increased β2-AR signaling in osteoblasts and subsequent suppression of bone formation and enhanced bone resorption. Moreover, mice lacking the glutamate transporter GLAST, which results in increased glutamate levels in the brain, exhibit a low bone mass phenotype, further supporting the catabolic role of central glutamate signaling in bone metabolism [61].

Peripheral glutamate signaling, on the other hand, has been shown to have both anabolic and catabolic effects on bone, with glutamate receptors (particularly NMDA receptors) expressed on osteoblasts and osteoclasts. In vitro studies have demonstrated that glutamate can stimulate osteoblast proliferation and differentiation through the activation of NMDA receptors, while also promoting osteoclastogenesis and bone resorption. The net effect of peripheral glutamate signaling on bone metabolism appears to be dependent on the concentration of glutamate and the specific cell type and receptor involved [62].

#### γ-Aminobutyric acid (GABA)

γ-Aminobutyric acid (GABA) is the primary inhibitory neurotransmitter in the CNS and has been implicated in the regulation of bone metabolism through both central and peripheral mechanisms [63].

Central gamma-aminobutyric acid (GABA) signaling is implicated in promoting bone anabolism, as evidenced by the correlation between elevated GABA levels in the brain and increased bone mass coupled with reduced osteoclast activity. This phenomenon is believed to be facilitated by the inhibitory effects of GABA on the sympathetic nervous system (SNS), resulting in diminished beta-2 adrenergic receptor (β2-AR) signaling in osteoblasts, thereby fostering bone formation and mitigating bone resorption. Notably, mice deficient in the GABAB receptor displays a phenotype characterized by decreased bone density, providing additional evidence for the anabolic impact of central GABA signaling on bone metabolism [64].

Peripheral GABA signaling has also been shown to have an anabolic effect on bone, with GABA receptors (particularly GABAB receptors) expressed on osteoblasts and osteoclasts [65]. In vitro studies have demonstrated that GABA directly stimulates osteoblast proliferation and differentiation while inhibiting osteoclastogenesis through the activation of GABAB receptors. Moreover, mice lacking GABAB receptors specifically in osteoblasts exhibit a low bone mass phenotype, highlighting the importance of peripheral GABA signaling in maintaining skeletal homeostasis [66].

These findings suggest that neurotransmitters plays a predominantly anabolic role in the regulation of bone metabolism, with both central and peripheral signaling pathways promoting bone formation and inhibiting bone resorption. Further research is needed to clarify the specific mechanisms underlying these effects and to explore the potential therapeutic implications of targeting neurotransmitters signaling in the context of skeletal disorders.

## Bone-derived factors influencing the CNS

While the CNS exerts significant control over bone metabolism, recent evidence suggests that the bone itself also secretes factors that can influence brain function and behavior. These bone-derived factors, also known as osteokines, have been shown to cross the blood–brain barrier and modulate various aspects of CNS function, including cognitive processes, memory, and mood. In this section, we will discuss two key bone-derived factors that have been implicated in the regulation of brain function: osteocalcin and fibroblast growth factor 23 (FGF23).

### Osteocalcin

Osteocalcin is a small, γ-carboxylated protein secreted by osteoblasts during bone formation and has traditionally been used as a marker of bone turnover. However, recent studies have revealed that osteocalcin also functions as a hormone, regulating glucose and energy metabolism, and influencing brain function and behavior [9].

Osteocalcin, particularly in its undercarboxylated form (ucOC), has been shown to cross the blood–brain barrier and bind to neurons in various regions of the brain, including the brainstem, midbrain, and hippocampus [10]. In the hippocampus, a key region involved in learning and memory, ucOC has been demonstrated to promote the expression of brain-derived neurotrophic factor (BDNF) and other synaptic proteins, thereby enhancing synaptic plasticity and improving cognitive function [67]. Mice lacking osteocalcin exhibit impairments in spatial learning and memory, as well as increased anxiety-like and depressive-like behaviors, highlighting the importance of osteocalcin in the regulation of brain function [10].

In addition to its effects on cognitive function, osteocalcin has also been implicated in the regulation of mood and stress response. ucOC has been shown to stimulate the synthesis of monoamine neurotransmitters, such as serotonin, dopamine, and norepinephrine, in the brain, which play crucial roles in the regulation of emotional states [68]. Mice lacking osteocalcin exhibit increased anxiety-like and depressive-like behaviors, which can be rescued by the administration of exogenous ucOC.

Clinical studies have provided further evidence for the role of osteocalcin in brain function. Serum levels of ucOC have been positively associated with cognitive performance and memory in elderly individuals, and reduced serum levels of ucOC have been observed in patients with Alzheimer's disease and depression [69]. These findings suggest that osteocalcin may serve as a potential biomarker for cognitive decline and neuropsychiatric disorders, and highlight the therapeutic potential of targeting osteocalcin signaling in the context of CNS disorders.

### FGF23

FGF23 is a bone-derived hormone that plays a central role in the regulation of phosphate and vitamin D metabolism. FGF23 is secreted primarily by osteocytes and osteoblasts in response to increased serum phosphate levels, and acts on the kidneys to promote phosphate excretion and suppress vitamin D synthesis [70]. While the primary function of FGF23 is to maintain phosphate homeostasis, recent evidence suggests that FGF23 may also influence brain function and behavior.

FGF23 has been shown to cross the blood–brain barrier and bind to FGF receptors (FGFRs) expressed in various regions of the brain, including the hippocampus, cortex, and hypothalamus [71]. In the hippocampus, FGF23 has been demonstrated to impair synaptic plasticity and cognitive function by reducing the expression of BDNF and other synaptic proteins [72]. Mice with elevated serum levels of FGF23 exhibit impairments in spatial learning and memory, as well as increased anxiety-like and depressive-like behaviors [73].

In addition to its effects on cognitive function, FGF23 has also been implicated in the regulation of neuroinflammation and oxidative stress in the brain. Elevated levels of FGF23 have been associated with increased expression of pro-inflammatory cytokines and reactive oxygen species in the hippocampus and cortex, which may contribute to the pathogenesis of cognitive impairment and neuropsychiatric disorders [74].

Clinical studies have contributed additional evidence supporting the involvement of FGF23 in brain function. Elevated levels of FGF23 in the serum have been linked to cognitive decline, dementia, and depression in individuals with chronic kidney disease, while increased levels of FGF23 have been detected in the cerebrospinal fluid of patients with Alzheimer's disease. These results indicate that FGF23 could potentially serve as a biomarker for cognitive impairment and neuropsychiatric conditions, underscoring the therapeutic opportunities associated with targeting FGF23 signaling in central nervous system disorders.

The discovery of osteocalcin and FGF23 as bone-derived factors influencing brain function has opened up new avenues for understanding the complex interactions between the skeletal and nervous systems [75]. These findings suggest that the bone is not merely a target for neural regulation, but also an active endocrine organ that can modulate brain function and behavior. Further research is needed to elucidate the specific mechanisms underlying the effects of osteokines on the brain, and to explore the therapeutic potential of targeting these signaling pathways in the context of CNS disorders.

### Bone morphogenetic proteins

Bone morphogenetic proteins (BMPs) are important growth factors that play key roles in the regulation of bone development and bone metabolism. In recent years, more and more evidence suggests that BMPs and their signaling pathways are closely related to various neurological diseases [76].

#### Neural development and differentiation

BMPs are involved in the development of central nervous system by regulating the proliferation, differentiation and apoptosis of neural stem cells [77]. It has been found that BMP2, BMP4 and BMP7 are expressed at different stages of embryonic brain development and regulate key events such as neural tube closure, neural crest formation, and neuronal and glial cell differentiation. Abnormalities in the signaling pathway of BMPs can lead to a series of neurodevelopmental disorders, such as spina bifida, microcephaly and other congenital diseases [78].

#### Neurodegenerative diseases

BMPs and their signaling pathways are abnormally regulated in neurodegenerative diseases such as AD and Parkinson's disease (PD) [79]. In brain tissues of AD patients and animal models, the expression of BMP2, BMP4 and BMP6 was up-regulated, while the expression of BMP9 was down-regulated, etc. BMPs can be involved in the pathogenesis of AD by regulating inflammatory response, oxidative stress, Aβ metabolism and other mechanisms [80]. For example, BMP4 can promote the transformation of microglia to inflammatory phenotype and aggravate the neuroinflammatory response, while BMP9 can inhibit the production and aggregation of Aβ and play a neuroprotective role [81]. In PD, the expression of BMP2 and BMP7 was down-regulated, and exogenous BMP7 administration was able to protect midbrain dopaminergic neurons and improve the motor function of PD animal models [82]. These findings suggest that the modulation of BMPs signaling pathway may provide new ideas for the prevention and treatment of neurodegenerative diseases.

#### Stroke

The BMPs signaling pathway plays a dual role in the damage response and repair process after ischemic and hemorrhagic strokes [83]. On the one hand, the rapid increase in BMP2, BMP4, and BMP7 expression after ischemia or hemorrhage can promote astrocyte proliferation, aggravate neuroinflammation and apoptosis, and amplify brain injury. On the other hand, BMP7 can promote nerve regeneration, enhance proliferation and differentiation of neural stem cells, and improve functional recovery after stroke [84]. Therefore, precise regulation of BMPs signaling is expected to inhibit the damage response in the acute phase of stroke and promote endogenous repair in the recovery phase.

#### Psychiatric disorders

Abnormalities in BMPs signaling pathway are associated with depression, schizophrenia, bipolar disorder and other mental diseases [85]. BMP6 levels in peripheral blood of patients with depression were reduced and negatively correlated with the severity of depressive symptoms [86]. In animal studies, chronic stress-induced microglia activation and neuronal damage through up-regulation of BMP4, leading to depressive behaviors, while BMP7 counteracted stress-induced neurodevelopmental abnormalities and depressive phenotypes [87]. In addition, several genomic studies in patients with schizophrenia and bipolar disorder have identified abnormalities in genes related to the BMP signaling pathway [88]. These evidences suggest that BMPs may be involved in the pathogenesis of psychiatric disorders by regulating neurodevelopment, inflammatory response and neuroplasticity.

In summary, BMPs from bone are crucial in the development of neurological diseases. Further research on their signaling pathways could lead to new treatments for conditions such as neurodegenerative diseases, stroke, and psychiatric disorders. However, the complexity of the BMPs signaling pathway in the nervous system requires more investigation.

### Lipocalin 2

Lipocalin 2 (LCN2), also known as neutrophil gelatinase-associated lipocalin (NGAL), is a lipid-transporting protein secreted by a variety of tissue cells, including bone tissues, and plays an important role in pathological processes, such as inflammatory responses, iron homeostasis regulation and organ damage [89]. In recent years, more and more studies have shown that LCN2 is closely related to a variety of neurological diseases, and the main evidences are as follows.

#### AD

Several studies have found that the levels of LCN2 in brain tissue, cerebrospinal fluid (CSF) and serum of AD patients are significantly higher than those of normal controls, suggesting that LCN2 may be involved in the pathogenesis of AD [90]. Brain histological studies showed that LCN2 accumulated abnormally in the brain tissues of AD patients, and co-localized with amyloid plaques and neurofibrillary tangles [91]. Animal experiments further confirmed that LCN2 promotes the development of AD through multiple mechanisms: (1) LCN2 activates toll-like receptor 4 (TLR4), induces microglia and astrocytes activation, causes the release of inflammatory factors and reactive oxygen species (ROS), and exacerbates neuroinflammation and oxidative stress damage; (2) LCN2 up-regulates the expression of amyloid precursor protein (APP) and β-secretase (BACE1), which is a key factor in the development of AD; (3) LCN2 up-regulates the expression of amyloid precursor protein (APP) and β-secretase (BACE1), which is a key component in the development of AD. (4) LCN2 up-regulates the expression of amyloid precursor protein (APP) and β-secretase (BACE1), and promotes the production and aggregation of β-amyloid (Aβ), accelerating the pathological process of AD; (5) LCN2 induces the over-phosphorylation of Tau protein through the promotion of iron accumulation and oxidative stress, and the formation of neurofibrillary tangles, which leads to neuronal damage [92]. On the contrary, knockdown of LCN2 gene or administration of LCN2-neutralizing antibody attenuated cognitive dysfunction, Aβ deposition and Tau pathology in AD mice [93]. Therefore, LCN2 may be an important mediator and potential therapeutic target in the pathogenesis of AD.

#### PD

Serum and cerebrospinal fluid levels of LCN2 were significantly elevated in PD patients and positively correlated with the degree of motor dysfunction, suggesting that LCN2 is involved in the pathogenesis of PD [94]. In PD animal model and dopaminergic neuron cell model, LCN2 induced inflammatory factors (e.g., TNF-α and IL-1β) and ROS production by activating the NF-κB pathway, causing neuroinflammatory responses and oxidative stress damage, and ultimately leading to apoptosis of dopaminergic neurons [95]. In addition, LCN2 may accelerate the pathological progression of PD by promoting α-synuclein aggregation and diffusion. Knockdown of LCN2 gene or blocking of LCN2 signaling could alleviate the motor deficits and dopaminergic neuron loss in animal models of PD, suggesting that LCN2 may be one of the therapeutic targets for PD.

#### Ischemic stroke

After cerebral ischemia, LCN2 expression was rapidly induced in peri-infarct tissues and increased with the extent of infarction and neurological deficit, suggesting that LCN2 is involved in ischemic injury [96]. Mechanistic studies have shown that LCN2 aggravates cerebral ischemic injury by promoting inflammatory response, oxidative stress and blood–brain barrier disruption: (1) LCN2 activates microglia and astrocytes, promoting inflammatory factors and ROS production, causing secondary injury; (2) LCN2 up-regulates matrix metalloproteinase 9 (MMP-9) activity, damaging the integrity of the blood–brain barrier and aggravating cerebral edema; (3) LCN2 activates iron accumulation in the peripheral tissues of infarcts and increases with the extent of infarcts and neurological deficits, suggesting its involvement in ischemic injury. LCN2 induces neuronal apoptosis by promoting iron accumulation, exacerbating lipid peroxidation and mitochondrial dysfunction [97]. Inhibition of LCN2 expression or activity can reduce the infarct volume and neurological deficits in cerebral infarction mice, prolong the treatment time window, and show its potential as a cerebroprotective target.

#### Mental diseases

LCN2 is abnormally expressed in depression, bipolar disorder, schizophrenia and other mental diseases [98]. LCN2 levels in serum, cerebrospinal fluid (CSF) and peripheral blood mononuclear cells (PBMCs) of depressed patients were significantly elevated and positively correlated with the severity of depressive symptoms. Chronic unpredictable stress and glucocorticoid administration induced elevated LCN2 expression and depressive-like behavior in rodents. Knockdown of LCN2 gene or administration of LCN2-neutralizing antibody alleviated depressive behavior, increased hippocampal neurogenesis, and improved stress responsiveness in mice [99]. Mechanistic studies suggest that LCN2 may cause neuroinflammation by activating NLRP3 inflammasome and inducing IL-1β precursor shearing, and lead to depressive symptoms by disrupting the hippocampal blood–brain barrier and affecting monoamine transmitter and trophic factor homeostasis. The peripheral blood levels of LCN2 in patients with bipolar disorder and schizophrenia are also disturbed, but the specific mechanism is not clear. In conclusion, LCN2 is closely related to many common psychiatric disorders and is expected to be a biomarker for assessing disease activity and prognosis.

In summary, LCN2, a bone-derived inflammatory mediator, is crucial in the development of neurological disorders like AD, PD, stroke, and psychiatric disorders through various mechanisms such as inflammation, oxidative stress, and disruption of iron homeostasis and the blood–brain barrier. It could serve as a new biomarker for disease severity, prognosis prediction, and treatment guidance. Targeting LCN2 with antibodies, antagonists, or small molecule inhibitors may offer promising therapeutic approaches for neurological diseases.

### Sclerostin

Sclerostin (SOST), a glycoprotein secreted by osteoblasts, is an important negative regulator of the Wnt/β-catenin signaling pathway and plays a key role in bone metabolism and skeletal diseases such as osteoporosis [100]. In recent years, more and more evidences have shown that SOST is closely related to a variety of neurological diseases, which are mainly reflected in the following aspects: Alzheimer’s disease (Alzheimer’s disease) and osteoporosis.

#### AD

CSF and serum SOST levels are significantly higher in AD patients than in normal controls, suggesting that SOST may be involved in the pathogenesis of AD [101]. Further studies showed that SOST accumulated abnormally in the brain tissue of AD patients and co-localized with pathological changes such as Aβ plaques and Tau protein phosphorylation. Animal experiments demonstrated that specific overexpression of SOST in AD transgenic mice exacerbated Aβ deposition, Tau protein phosphorylation and neuronal loss, and accelerated the deterioration of cognitive function [102]. On the contrary, administration of SOST-neutralizing antibody or knockdown of SOST gene could activate the Wnt/β-catenin pathway and alleviate the cognitive impairment and neuropathological changes in AD mice. Mechanistic studies suggest that SOST may be involved in the pathogenesis of AD through the following pathways: (1) SOST inhibits neural stem cell proliferation and differentiation by antagonizing the Wnt/β-catenin pathway, thus hindering nerve regeneration and synaptic plasticity; (2) SOST increases the activity of β-secretase (BACE1), accelerating the production and aggregation of Aβ; and (3) SOST activates GSK-3β to promote the hyperphosphorylation of Tau protein, causing neurological deficits and neuropathological alterations. SOST activates GSK-3β, which promotes Tau protein hyperphosphorylation and causes neurofibrillary tangles; (4) SOST induces oxidative stress and mitochondrial dysfunction, leading to neuronal apoptosis [103]. Therefore, SOST may be a risk factor and a therapeutic target for AD.

#### Vascular cognitive impairment

Vascular cognitive impairment (VCI) is a cognitive dysfunction caused by cerebrovascular lesions, and is one of the main types of dementia in old age [104]. Clinical studies have found that serum SOST levels in patients with VCI are significantly higher than those of normal controls, and are positively correlated with the degree of cognitive impairment. Animal experiments further confirmed that SOST expression was significantly elevated in the hippocampus and cortex in the rat VCI model induced by bilateral common carotid artery ligation (2VO), and the administration of SOST-neutralizing antibody significantly improved the learning memory and spatial cognitive abilities of rats [105]. Mechanistic studies showed that SOST may aggravate the cognitive impairment of VCI in the following ways: (1) SOST inhibits the proliferation of cerebral microvascular endothelial cells and neovascularization by blocking the Wnt/β-catenin pathway, thus aggravating cerebral ischemia–hypoxia injury; (2) SOST promotes the calcification of vascular smooth muscle cells and the formation of plaques by activating the GSK-3β and NF-κB pathways, thus accelerating the cerebral arteriosclerosis; (3) SOST promotes the calcification and plaque formation of cerebral arteries by activating GSK-3β and NF-κB pathways. SOST increases vascular permeability, destroys the integrity of the blood–brain barrier, and leads to aggravation of neuroinflammation and oxidative stress. Therefore, SOST may be an important mediator in the pathogenesis of VCI, and the treatment of SOST is expected to improve the prognosis of VCI.

#### Ischemic stroke

After cerebral ischemia, the expression of SOST was rapidly up-regulated in peri-infarct tissues and increased with the infarct extent and neurological deficit, suggesting that SOST was involved in ischemic brain injury [106]. In a rat model of cerebral infarction induced by middle cerebral artery embolization (MCAO), administration of SOST-neutralizing antibody activated the Wnt/β-catenin pathway, reduced the size of infarct foci, and improved the recovery of neurological function. Mechanistic studies showed that SOST may aggravate ischemic brain injury by: (1) SOST antagonizes the Wnt/β-catenin pathway, inhibits neural stem cell proliferation and differentiation, and hinders nerve regeneration and axon germination; (2) SOST induces apoptosis of cerebral endothelial cells in the brain microvessels and disrupts the blood–brain barrier, thus aggravating cerebral edema; and (3) SOST activates the TLR4/NF-κB pathway, inducing inflammatory factors and reactive oxygen clusters, which are the most important factors in the development of the brain. SOST activates the TLR4/NF-κB pathway and induces the release of inflammatory factors and reactive oxygen species, leading to secondary brain injury [107]. Therefore, inhibiting the expression or activity of SOST may be a new strategy to reduce cerebral infarction injury and promote neural repair.

#### Multiple sclerosis

Multiple sclerosis (MS) is a chronic inflammatory demyelinating disease characterized by scattered demyelinating plaques and axonal degeneration in the central nervous system [108]. Clinical studies have found that CSF and serum SOST levels in patients with MS are significantly higher than those in normal controls, and are positively correlated with the severity of the disease and the degree of disability. Animal experiments further demonstrated that in a mouse model of experimental autoimmune encephalomyelitis (EAE), SOST was significantly elevated along the course of the disease, and the administration of SOST-neutralizing antibody could delay the onset of EAE and reduce the inflammatory infiltration and demyelinating lesions [109]. Mechanistic studies suggest that SOST may be involved in the pathogenesis of MS in the following ways: (1) SOST antagonizes the Wnt/β-catenin pathway, inhibits the proliferation and differentiation of oligodendrocyte precursor cells (OPCs) and regeneration of myelin sheaths, and hinders the repair of demyelination; (2) SOST induces the differentiation of Th17 cells and the secretion of inflammatory factors, such as IL-17, IFN-γ and other inflammatory factors, and aggravates the autoimmune response of the CNS; (3) SOST induces the differentiation of Th17 cells, and secretion of inflammatory factors, such as IL-17, IFN-γ and others, and aggravates the autoimmune reaction of the CNS. SOST induces Th17 cell differentiation and secretion of inflammatory factors such as IL-17 and IFN-γ, which exacerbate CNS autoimmune responses; and (4) SOST causes axonal degeneration and neuronal loss by up-regulating the RhoA-ROCK pathway. Therefore, SOST may be a key factor in the pathogenesis of MS, and immunotherapy targeting SOST is expected to be a novel therapy for MS.

In conclusion, SOST acts as a natural inhibitor of the Wnt/β-catenin pathway and is crucial in the development of neurological diseases such as Alzheimer’s, vascular cognitive impairment, stroke, and multiple sclerosis by hindering neurogenesis, promoting Aβ and Tau buildup, and triggering inflammation and axonal degeneration. SOST could be a biomarker for disease activity and prognosis in MS, and targeting it with immunotherapy may be a new treatment approach. However, more research is needed to understand how SOST functions at different disease stages and its interactions with other risk factors.

#### Others

Osteopontin (OPN), Osteoprotegerin (OPG), Periostin (POSTN) and Dickkopf-1 (DKK1) are newly discovered bone-derived factors associated with neurological disorders, which have been shown to play important roles in various neurodegenerative diseases, cerebrovascular diseases and neurological injuries [110].

OPN is a phosphorylated glycoprotein that is expressed in osteoblasts, macrophages and activated T cells, and is involved in bone remodeling, inflammation and tissue repair, etc. [111]. OPN is found throughout the central nervous system and is involved in neurodevelopment, synaptic remodeling, neuroglial interactions, and blood–brain barrier regulation. It is linked to neurodegenerative and inflammatory diseases such as AD, PD, ALS, and MS, potentially impacting disease progression through its role in inflammation, oxidative stress, blood–brain barrier integrity, and Aβ clearance [112]. It is expected to become a biomarker for the diagnosis and prognosis of neurological disorders, and to provide a new potential target for therapeutic treatment.

OPG is a member of the soluble tumor necrosis factor receptor superfamily, which is expressed in osteoblasts and inhibits osteoclast differentiation and bone resorption by blocking the binding of RANKL to the osteoclast membrane surface receptor RANK [113]. OPG in the central nervous system helps regulate neuronal survival, synapse formation, axon growth, and myelin sheath formation. It has neuroprotective effects in Alzheimer’s disease, Parkinson’s disease, and cerebral ischemia by preventing neuronal apoptosis, oxidative stress, and inflammation, and maintaining synaptic stability [114]. Therefore, OPG is expected to be a new therapeutic strategy for neurodegenerative diseases and cerebrovascular diseases.

POSTN is a secreted extracellular matrix protein that is expressed in osteoblasts, fibroblasts and smooth muscle cells, and is involved in tissue development and injury repair [115]. POSTN is involved in the migration and differentiation of neural crest cells in the embryonic stage, as well as the regeneration of peripheral nerves and repair of damage to the central nerves in the adult body. POSTN expression was found to be reduced in cerebrospinal fluid and serum of AD patients, and correlated with the degree of cognitive dysfunction, and POSTN-deficient mice showed accelerated aging, decreased learning and memory ability, and reduced hippocampal neuron density [116]. In addition, POSTN expression was up-regulated after traumatic brain injury and improved cognitive and motor function recovery by promoting astrocyte migration, vascular regeneration and synaptic remodeling. Therefore, POSTN may be a potential marker for the diagnosis and prognostic evaluation of TBI and AD, and also provides a new target for neuroprotection and promotion of repair.

DKK1 is an important antagonist of the Wnt pathway and is expressed in osteoblasts and mesenchymal stem cells, inhibiting osteogenic differentiation and bone formation by blocking Wnt/β-catenin signaling. DKK1 is closely related to AD, and its expression is elevated in the cerebrospinal fluid, serum, and brain tissues of patients with AD and is positively correlated with the pathology of Aβ and Tau, as well as with the cognitive impairment. DKK1 inhibits synaptic synapses by blocking the Wnt pathway. DKK1 inhibits synaptogenesis and plasticity by blocking the Wnt pathway, induces synaptic loss and cognitive deficits, and exacerbates AD pathology by activating GSK3β and up-regulating β-secretase and Tau phosphorylation. Blockade of DKK1 attenuated AD-like cognitive deficits and neuropathological changes [117]. Therefore, DKK1 may be a risk factor and a therapeutic target in the development of AD, and immunotherapy targeting DKK1 is expected to delay the progression of AD.

In conclusion, bone-derived factors such as OPN, OPG, POSTN, and DKK1 regulate the development, homeostasis, and damage repair of the nervous system through various pathways, and play important roles in neurodegenerative diseases, cerebrovascular diseases, and traumatic brain injury. Elucidating the mechanism of these factors and developing specific regulatory drugs are expected to bring new breakthroughs in the precise diagnosis and treatment of neurological diseases. Meanwhile, these factors are closely related to bone metabolism, suggesting that there exists a complex bidirectional regulatory network between bones and the nervous system, and that disorders of the bone–brain axis may be the common pathological basis of neurological disorders, so systematic research is urgently needed to elucidate their mechanisms and guide disease prevention.

## Therapeutic implications and future directions

The brain–bone axis holds great promise for the development of novel therapeutic strategies targeting both skeletal and neurological disorders. Modulating the activity of the sympathetic nervous system, targeting hypothalamic neuropeptides, and harnessing the potential of bone-derived factors, such as osteocalcin and FGF23, may provide new approaches for the prevention and treatment of conditions such as osteoporosis, fractures, Alzheimer's disease, and depression. However, further research is needed to fully understand the specific mechanisms underlying the neural regulation of bone metabolism and the effects of bone-derived factors on brain function. Future studies should focus on identifying the specific neuronal populations and circuits involved in the regulation of bone metabolism, exploring the potential side effects of targeting these pathways, and establishing the long-term safety and efficacy of novel therapeutic approaches. In addition, the potential implications of the brain–bone axis for other physiological and pathophysiological processes, such as glucose metabolism and cardiovascular function, should be explored. As we continue to unravel the mysteries of this complex system, we may be able to unlock new strategies for improving both skeletal and neurological health.

## Conclusion

The brain–bone axis represents a fascinating and rapidly evolving field of research that has unveiled the intricate bidirectional communication between the central nervous system and skeletal metabolism. This review has explored the complex mechanisms, key players, and potential clinical implications of this crosstalk, highlighting the roles of neural pathways, hypothalamic neuropeptides, neurotransmitters, and bone-derived factors in the regulation of bone metabolism and brain function. The discovery of the brain–bone axis has opened up new avenues for understanding the pathophysiology of skeletal and neurological disorders and has the potential to lead to the development of novel diagnostic tools, therapeutic interventions, and preventive strategies that target both skeletal and neurological health. However, many questions remain unanswered, and further research is needed to fully elucidate the intricacies of this complex system.

## Data Availability

No datasets were generated or analysed during the current study.
